# A narrow QRS gallop rhythm

**DOI:** 10.1007/s12471-015-0654-0

**Published:** 2015-01-28

**Authors:** Daniël Mol, Andrew Tjon Joek Tjien, Jonas S.S.G. de Jong

**Affiliations:** Department of cardiology and cardiac surgery, Onze Lieve Vrouwe Gasthuis, Oosterpark 9, 1091 AC Amsterdam, The Netherlands

## Answer

The QRS complexes 1, 2, 4, 5 and 7 are not preceded by atrial activation. The QRS morphology is slightly different from an A-V conducted beat (QRS 3, 6). Therefore an origin in the bundle branch system is presumed for beats 1, 2, 4, 5 and 7. An escape beat (QRS 2, 5) is retrogradely conducted to the atrium with atrial activation followed by AV conduction and ventricular capture (#3, 6). This phenomenon has been described previously as escape-echo bigeminy [1]. This echo beat could be the result of dual AV nodal pathway physiology where the retrograde conduction goes through the slow pathway. Escape beats 1, 4 and 7 are not conducted antegradely after retrograde atrial activation. VA conduction of this beat results in slightly earlier atrial activation, possibly through a fast retrograde AV nodal pathway. Normal sinus rhythm recovered and the patient was discharged. However, a week later, the patient returned to the emergency department with symptomatic bradycardia, sinus arrest and chronotropic incompetence and a pacemaker was implanted. Electrophysiological study performed with the pacemaker did not show evidence of dual AV nodal physiology.

**Fig. 1:**
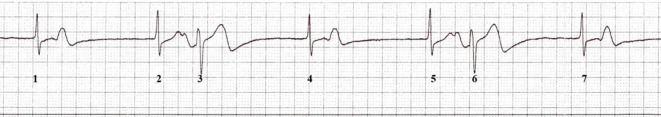
ECG recording during surgical pulmonary vein isolation

Sinus node dysfunction during surgical pulmonary vein isolation can result from different mechanisms: ablation near the vagal nerve can evoke a vagal response; ablation near the sinus node can inadvertently damage or electrically isolate the sinus node; ablation near the sinus node artery can result in sinus node ischaemia. In our patient a computed tomography angiogram revealed that the sinus node artery was a side branch of the circumflex coronary artery (Fig. 1).
